# Strengthening Community Responses to Economic vulnerability (SeCuRE): a protocol of an HIV status-neutral pilot randomized clinical trial with transgender women of color in Detroit, Michigan

**DOI:** 10.1186/s40814-024-01558-5

**Published:** 2024-11-08

**Authors:** Kristi Gamarel, Larissa Jennings Mayo-Wilson, Laura Jadwin-Cakmak, Lilianna Reyes, Dior’ Monro, Ini-Abasi Ubong, Stephen Sullivan, Julisa Abad, Jeynce Poindexter, Harmony Harris, Chanel Riser, J. Stephenson, Gabi Ortiz, Sarah M. Peitzmeier, Torsten B. Neilands, Tonia Poteat

**Affiliations:** 1 Department of Health Behavior and Health Equity, University of Michigan School of Public Health, Ann Arbor, MI 48109 USA; 2 Department of Health Behavior, University of North Carolina-Chapel Hill, Chapel Hill, NC USA; 3 Trans Sistas of Color Project, Detroit, MI USA; 4 Fair Michigan Foundation, Detroit, MI USA; 5 Boston College School of Social Work, Boston, MA USA; 6 Department of Health Behavior and Biological Sciences, University of Michigan School of Nursing, Ann Arbor, MI USA; 7 Department of Medicine, University of California, San Francisco, San Francisco, CA USA; 8 Duke University School of Nursing, Durham, NC USA

**Keywords:** Pilot, Microeconomic intervention, Transgender women, HIV prevention, HIV care

## Abstract

**Background:**

In the United States (US), transgender women of color experience cyclical, interlocking systems of structural and institutional oppression rooted in racism and transphobia, which fuel economic vulnerability. Together, cycles of intersecting racism, transphobia, and economic vulnerability create conditions that give rise to extreme HIV inequities among transgender women of color. Microeconomic interventions — designed to improve financial standing by increasing income generation and access to financial resources through entrepreneurship, cash transfers, and training — have the potential to address structural factors underlying HIV inequities. Over the past few years, several trans-led organizations, including the Trans Sistas of Color Project, have integrated microeconomic strategies, specifically emergency assistance, into their programming. The aim of the current study is to conduct a pilot randomized controlled trial (RCT) to evaluate the feasibility and acceptability of a definitive subsequent RCT and explore initial evidence of an enhanced microeconomic intervention to increase income generation and improve HIV prevention and care continua outcomes.

**Methods:**

This is a two-arm waitlist randomized controlled trial in which transgender women of color will be randomly allocated to either usual care that includes the Trans Sistas of Color Project’s existing microeconomic interventions, which includes the following: (1) US $250 in emergency assistance and (2) peer support to obtain legal gender affirmation (i.e., legal name and gender markers on identification documents) or the enhanced microeconomic intervention that includes usual care and will be enhanced to include the following: (1) 12 weekly educational group sessions on economic empowerment (i.e., job acquisition, income generation through micro-business, and financial literacy) and HIV prevention and care, (2) employment-focused mentoring, and (3) an unconditional grant (US $1200) for use towards acquiring self-led or formal employment. Participants in each condition will complete a baseline survey prior to randomization, a follow-up survey immediately following intervention completion, and 3-month survey after intervention completion. Participants will also complete qualitative exit interviews within 1 month of intervention completion for both conditions.

**Discussion:**

This study will be one of the first US-based pilot randomized clinical trials that builds upon existing community-led solutions to economic vulnerability to address HIV inequities. Findings will provide the necessary groundwork to examine intervention effectiveness in a future large-scale trial.

**Trials registration:**

NCT06212544.

**Protocol version:**

September 25, 2024, version 2.

**Supplementary Information:**

The online version contains supplementary material available at 10.1186/s40814-024-01558-5.

## Background

Transgender (trans) women are disproportionately affected by the HIV epidemic [1]. In the United States (US), evidence indicates that approximately 19–21% of trans women are living with HIV [2–4]. There are notable racial/ethnic inequities in HIV in which Black, Latina, and other trans women of color represent the majority of the cases among trans women [5–7]. Accumulating evidence documents inequities in HIV prevention and care continua outcomes among trans women of color [8, 9]. For example, evidence suggests that trans women of color have a 40% lower odds of lifetime HIV testing and 50% lower odds of HIV testing in the past 12 months compared with sexual minority cisgender men [10]. Studies also indicate very low pre-exposure prophylaxis (PrEP) uptake among PrEP-eligible trans women [11, 12] and low rates of virologic control among trans women living with HIV [8, 13–22]. Thus, interventions are needed that address inequities across both the HIV prevention and care continua among trans women of color women in the US.

Economic vulnerability, characterized by unemployment, limited financial resources, income insecurity, and unstable housing, is recognized as a structural determinant of HIV inequities. Due to the convergence of structural racism and transphobia, trans women of color experience high rates of income insecurity [23–27], unstable housing [23, 26–30], and unemployment [25, 27, 29, 31]. Unemployment rates among trans women of color have been estimated to be nearly four times higher than the national average [32], with many trans women living below the poverty line [25, 32] and reporting unstable housing in their lifetime [32, 33]. Research suggests that persons experiencing economic hardship have challenges prioritizing HIV prevention and care in the face of needing to meet basic needs (i.e., housing, food), resulting in lower rates of HIV testing [34], PrEP initiation [35], antiretroviral therapy (ART) adherence, and engagement in HIV care [36]. Additional evidence demonstrates that over a third of trans women of color have not been able to have their name or gender marker changed due to financial costs, a significant barrier to seeking work and accessing sexual health services [32]. In the US, state and federal legal identification documents are required to access education, employment, housing, and health care facilities, including HIV and related PrEP/ART services [37]. Trans women of color who lack legal gender affirmation are at greater risk for economic vulnerabilities [38, 39], which further fuel inequities across the HIV prevention and care continuum [40].

Microeconomic interventions have been shown to increase HIV prevention and care outcomes in global contexts by reducing economic drivers of HIV inequities and by increasing access HIV services. However, a dearth of microeconomic studies has been conducted among trans people in the US. Microeconomic interventions are designed to improve financial status by increasing entrepreneurship, savings, and/or employment, thereby addressing the structural factors underlying HIV inequities among economically marginalized individuals. Microeconomic strategies have included business loans, personal savings accounts, microgrants, vocational training, financial and business training, insurance provision, career planning, and mentoring [41]. Due to pervasive systemic barriers, traditional employment options may not always be considered viable poverty alleviation strategies for many trans women of color [42, 43]. Owning assets (i.e., microenterprise) has the potential to give trans women of color a sense of stability and enable them to expand their vision of possibility and health-promoting opportunities [44, 45]. Microeconomic interventions can also extend beyond income generation and focus on enhancing skills (i.e., industriousness, perseverance, persistence) that support long-term sexual health promotion, medication adherence, and job acquisition and retention [41].

Despite alarming rates of racism, transphobia, and economic vulnerability, it is important to underscore that trans women of color are resourceful and find creative ways to leverage existing community and individual assets to address racism, transphobia, and economic vulnerability in their communities [46–49]. In 2015, the Trans Sistas of Color Project (TSoCP) launched a mutual aid fund that consists of emergency assistance, a no-strings-attached (i.e., unconditional) microgrant, for trans women of color in Detroit, MI, US. During the COVID-19 pandemic, many other US-based trans-led organizations have found ways to support their communities through the provision of mutual aid and organized advocacy for justice [50, 51]. Additionally, several trans-led organizations, including board members of the Trans Sistas of Color Project, have formed legal partnerships that provide counsel in obtaining legal gender affirmation [52].

Our team conducted a formative program evaluation of TSoCP’s emergency assistance program available to all trans women of color in Detroit, Michigan. Between November 2020 and February 2021, emergency assistance was provided to 30 trans women of color, and all recipients received their requested amounts which ranged from US $80 to US $500 (*M* = US $200, *SD* = US $140). Those who requested funds were invited to participate in a brief mixed-methods evaluation in which eight trans women of color agreed to participate in a brief baseline (pre-grant receipt) and 1-month follow-up (post-grant receipt). Qualitative analyses from the follow-up identified specific desired strategies to augment TSoCP’s program, including grants to use towards self-led or formal employment, education and skill building for self-led or formal employment, and mentorship [53]. Thus, the *Strengthening Community Responses to Economic vulnerability (SeCuRE)* project was designed to build on existing community-led services to reduce economic vulnerabilities and HIV inequities among trans women of color in the US through employment-focused microgrant support, improved financial literacy, and employment-focused mentoring.

### Theoretical frameworks

This pilot study is guided by two theoretical frameworks: (1) The gender affirmation model and (2) asset theory. *The gender affirmation model* is a conceptual framework that outlines how intersectional forms of oppression contribute to inequities in HIV among trans women of color. Gender affirmation refers to ways in which trans women are affirmed in their gender identity and expression and has been described as a key social determinant of trans health [42, 54]. Dimensions of gender affirmation include legal (e.g., legal document changes to name and gender marker), medical (e.g., hormone therapy), social (e.g., use of correct pronouns), and psychological (e.g., internalized affirmation) [42, 54]. Obtaining gender affirmation contributes to positive identity development, self-esteem, and safety [54, 55]. However, trans women of color whose needs for gender affirmation are not met due to systemic racism, transphobia, and economic vulnerability may seek gender affirmation in ways that place them in unsafe situations (e.g., high-risk sex work, reliance on abusive partners, or condomless sex) [54]. Additionally, trans women of color who lack legal gender affirmation are at heightened risk for economic vulnerability [38, 39], which fuels HIV inequities [27, 40]. Peer-delivered interventions and mentorship have been identified as important among trans women of color as community members with shared identities are critical in the provision of social and psychological gender affirmation, in that they can offer safety, trustworthiness, and mutual collaboration [56]. Community mentors are also primary and trusted sources of information about income generation, navigating employment and other systems as a trans woman, legal and medical gender affirmation, PrEP, ART adherence, and other sexual health promotion strategies [57, 58].

*Asset theory* is a conceptual model positing that increases in productive assets (i.e., business, rental property) can influence individual behavior by motivating health-promoting attitudes and behaviors to avoid negative consequences [59, 60]. Such assets may also minimize economic constraints and stressors that place individuals in vulnerable contexts [59, 60]. Thus, peer-led HIV status-neutral microeconomic interventions that build on existing community strengths to increase income generation and access to legal, social, and psychological gender affirmation hold high promise to reduce HIV inequities among trans women of color.

**Fig. 1:**
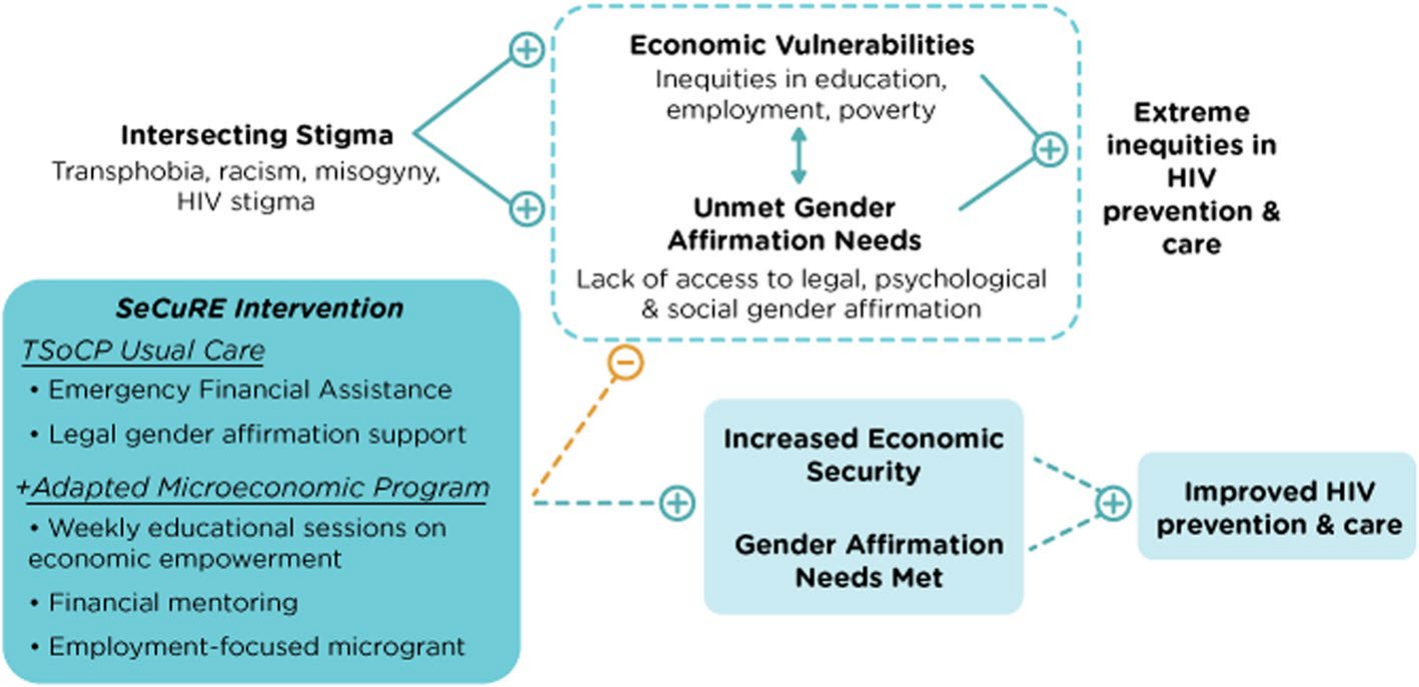
Conceptual framework for the SeCuRE intervention

Figure 1 includes our conceptual framework and highlights the key mechanisms of change. First, based on the gender affirmation model, we posit that trans women of color experience inequities in HIV prevention and care continua outcomes as a result of intersectional stigma, which results in economic vulnerability and unmet gender affirmation needs. Second, based on TSoCP’s existing programming, we posit that our peer-delivered *SeCuRE* intervention will enhance access to legal, psychological, and social gender affirmation for trans women of color, which will result in improved HIV prevention and care continua outcomes. Third, based on asset theory, we propose that our *SeCuRE* intervention will equip trans women of color with assets and skills necessary for income generation, which in turn will improve HIV prevention and care continua outcomes. Taken together, our *SeCuRE* intervention seeks to provide economically vulnerable trans women of color with financial assistance coupled with individualized employment-focused mentorship and peer-led, group-based sessions to build knowledge and skills related to financial literacy, income generation (i.e., self-led or formal employment), and gender-affirming sexual health promotion to address inequities in HIV prevention and care continua outcomes.

This paper outlines the protocol for a pilot study of the *SeCuRE* intervention: a 12-week enhanced microeconomic intervention that builds upon TSoCP’s existing emergency assistance and legal gender affirmation programs and incorporates promising components of a US-based microenterprise intervention designed for Black young adults in Baltimore, MD, US [41, 61, 62].

## Methods

### Aims and objectives

The aims of the current study are to establish feasibility and acceptability of the *SeCuRE* intervention to improve HIV prevention and care continua outcomes. To meet this aim, the study has the following objectives:

1. To deliver a two-armed pilot RCT of the *SeCuRE* intervention with 40 trans women of color
2. To determine the feasibility and acceptability of the intervention and research procedures with trans women of color

### Trial design

This study is a two-armed pilot RCT in which trans women of color were randomized to the immediate intervention or a waitlist control group. Participants were randomized into one of two conditions: (1) the immediate intervention arm that includes the 12-week *SeCuRE* microeconomic intervention in addition to TSoCP’s usual care (UC), which includes a one time payment of US $250 in emergency assistance and access to the Fair Michigan Name Change Clinic that includes support for legal name/gender marker change on identity documents, and (2) a waitlist control arm in which participants will receive UC and then will be offered the *SeCuRE* intervention after the 3-month comparison follow-up period. The protocol was developed in accordance with the Standard Protocol Items: Recommendations for Interventional Trials (SPIRIT) 2013 checklist and in collaboration with community partners who provided feedback on TSoCP’s existing programs and a promising microeconomic intervention designed with Black young adults in Baltimore [41, 61, 62] and who adapted the intervention components.

### Setting

The study takes place at the Ruth Ellis Center located in Detroit, MI, US. The Ruth Ellis Center is a community center that serves economically vulnerable sexual and gender minority youth and young adults of color. The Ruth Ellis Center also hosts social events and serves as a safe space where many trans people of color regardless of their age can get their basic needs met (e.g., food, linkage to community resources) and socialize with peers and role models in their community. The Ruth Ellis Center is the fiduciary of the Trans Sistas of Color Project and provides a space for the Fair Michigan’s Name Change Clinic.

### Eligibility criteria

To be eligible to participate, trans women needed to self-report being as follows: (1) at least 18 years old; (2) assigned male sex at birth; (3) self-identify as female, transgender woman, or another feminine gender identity; (4) self-identify as a person of color (i.e., any racial/ethnic identity except non-Hispanic White); (5) reports earning less than US $32,800 gross annual income (current living wage in Michigan); (6) reports condomless sex in the past 6 months; (7) lives in Detroit, MI, US, greater metropolitan area (~ 50 mile radius); and (8) speaks English.

### Interventions

The experimental *SeCuRE* intervention consists of a 12-week program in addition to UC. The *SeCuRE* program includes the four following components. First, participants receive 12 weekly microeconomic sessions lasting 2 h each and are conducted in-person. Session topics focus on income acquisition assistance through skill development and goal setting relating to job seeking (i.e., resumes, job application, interviewing), income generation through micro-business (i.e., self-employment, entrepreneurship, accessing clients, making a profit), and financial literacy (i.e., budgeting, managing credit). To address the relationship between economic vulnerability and HIV inequities, session topics also focus on knowledge and uptake of biomedical HIV prevention and treatment strategies (i.e., use of PrEP/ART) with particular attention to discussion of participants’ financial constraints and trade-offs to protecting against HIV and how safer income generation can be leveraged to improve HIV outcomes (i.e., paying for travel to PrEP/ART clinics, acquiring higher-paying employment or health insurance, or refusing condomless sex work). Peer health educators (PHEs) co-facilitate the interactive group sessions, including role-play, discussions, games, and demonstrations. Each session prioritizes gender-affirming principles of social cohesion, mutual support, and identify affirmation. Second, participants receive employment-focused mentorship where participants meet with an employment-focused mentor weekly (~ 30 min/week) to assist them in acquiring or maintaining employment, initiating or expanding self-led income generation, or navigating financial decisions, including planning for use of their study-provided microgrants of US $1200. We identified approximately eight mentors, and each mentor is provided with an honorarium of US $450 per mentee. Each participant is provided with US $1200 to use to support income generation — either in engaging in self-led employment (i.e., purchasing business supplies, marketing, communication, and travel for selling handmade goods and services) or in engaging in formal employment (i.e., traveling to job interview, paying for licensure or skills course, paying for gender transition supports to reduce employment discrimination). With their mentors, participants develop an individualized *SeCuRE* income generation plan that outlines how they will spend their microgrant.

Participants randomized to the control condition receive UC during the 12-week period following randomization. After the RCT follow-up period is complete, waitlist control participants are offered delayed access to the same *SeCuRE* intervention that is provided immediately to intervention arm participants.

### Outcome

Our primary outcome is feasibility and acceptability indices assessed during intervention implementation, follow-up surveys, and exit interviews.

### Participant timeline

**Fig. 2:**
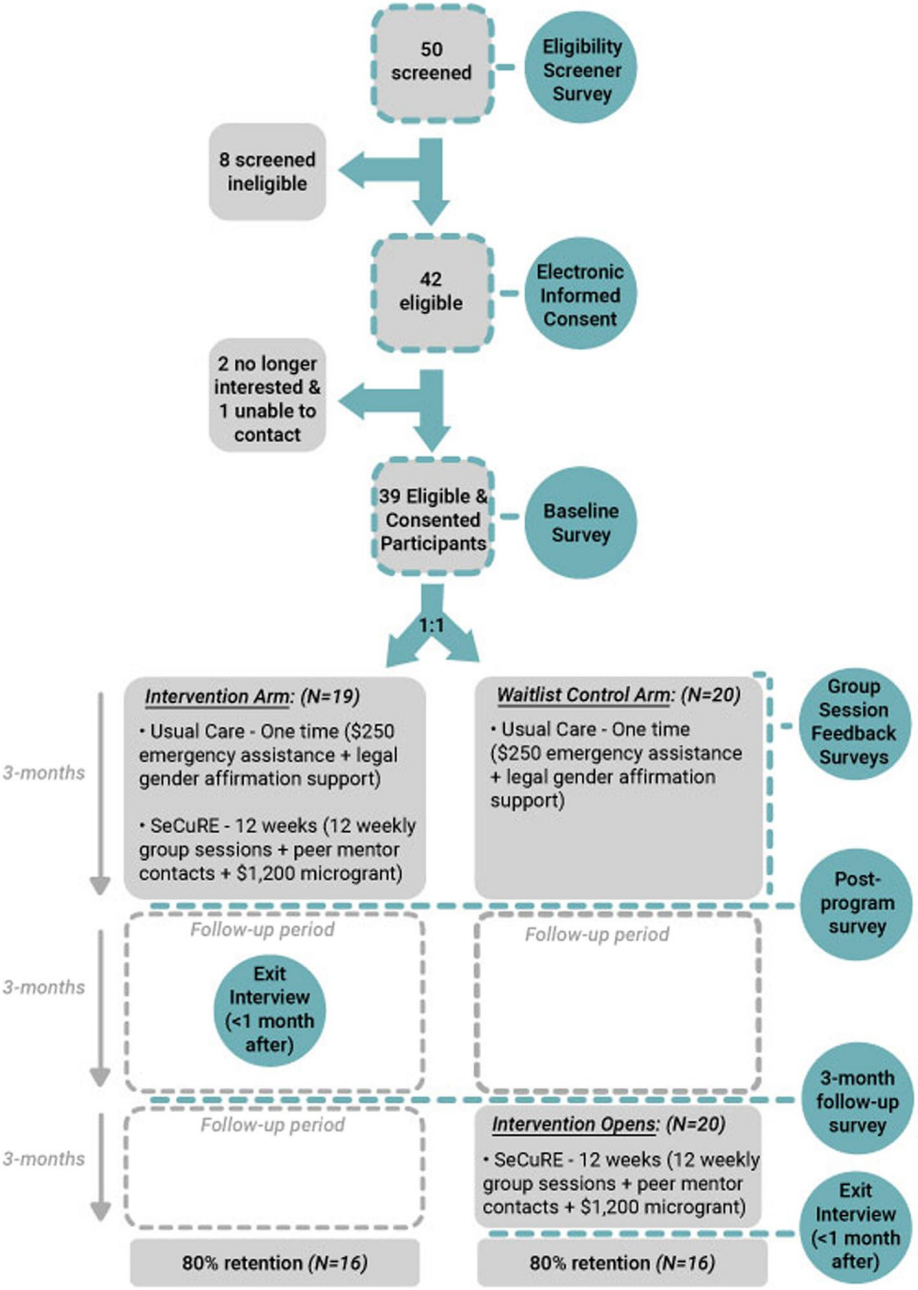
Study flow chart

See Fig. 2 for a chart of the study flow and timeline. To date, we have screened 50 participants with 42 eligible and 39 enrolled and randomized (*n* = 19 immediate intervention; *n* = 20 waitlist control). Each condition received survey assessments at baseline prior to randomization and will receive survey assessments post-intervention following the intervention arm’s completion of the 12-week SeCuRE intervention and 3 months after the intervention arm’s completion of the SeCuRE intervention (approximately 24 weeks after completion of the baseline survey). Additionally, qualitative exit interviews will be conducted within 1 month of completion of the SeCuRE intervention for both the immediate intervention and waitlist control conditions.

### Sample size

Although the main purpose of this study is to determine preliminary feasibility and acceptability and to visualize changes in outcomes and mechanisms of change over time rather than to perform formal hypothesis tests, we conducted several power analyses using NCSS PASS 21 [63] to supply additional information. We aimed to enroll 40 participants and ultimately enrolled 39 participants. Assuming power = 0.80, *α* = 0.05, and *N* = 32 participants available at the last follow-up, we computed confidence interval widths for (1) proportions for binary variables and (2) means for continuous variables measuring feasibility and acceptability. For example, for the study enrollment proportion, assuming *α* = 0.05, power = 0.80, and an 80% enrollment benchmark, the width of the confidence interval for single proportions is 29.8% (standardized distance to the limit: 0.40). For continuous variables, the distance from the mean to the confidence limit is 0.36. For mixed models proposed below to explore group differences in outcomes, the minimum detectable proportion increase in HIV care and prevention ranged from 33.7 to 42.8% (standardized effect size *h* = 0.75–0.92; odds ratios = 4.94–7.56). Taken collectively, this study is powered to detect small to medium distances to confidence limits for descriptive statistics to assess feasibility and acceptability (primary goal) and large effects for preliminary effectiveness effects (secondary goal), though, as noted above, formal hypothesis testing will not be the focus of this pilot study.

### Recruitment

We employed a multipronged outreach strategy to recruit trans women of color, including the following:

1. Online recruitment: Banner ads and posts will be placed on social media platforms used by trans women of color (Facebook, Instagram).
2. Print ads: Flyers will be placed in healthcare and social service agencies in Detroit.
3. Outreach: Our study team conducted outreach in areas where trans women of color congregate in Detroit.

Recruitment materials provided a study phone number or email to contact the study team about interest in participating. A study team member contacted interested individuals by phone, shared additional information about the study, and answered any questions they may have. The study team member then asked the interested individual if they would like to take a brief survey to see if they are eligible for the study. If eligible, the potential participant needed to complete a set of enrollment activities (Table 1, Fig. 2). The enrollment process consisted of obtaining informed consent, completing a baseline survey, and randomization. These activities were completed in person at Ruth Ellis or at-home remotely. Eligible potential participants who indicate they are interested in joining the study completed an informed consent with a study team member either remotely by phone or video call or in-person in a private location at the Ruth Ellis Center. If a potential participant was not eligible or chose not to consent, they were thanked for their time and were not be able to proceed with the enrollment process. Eligible trans women who chose to consent were able to complete the online baseline survey either at home or in-person.

**Table 1:**
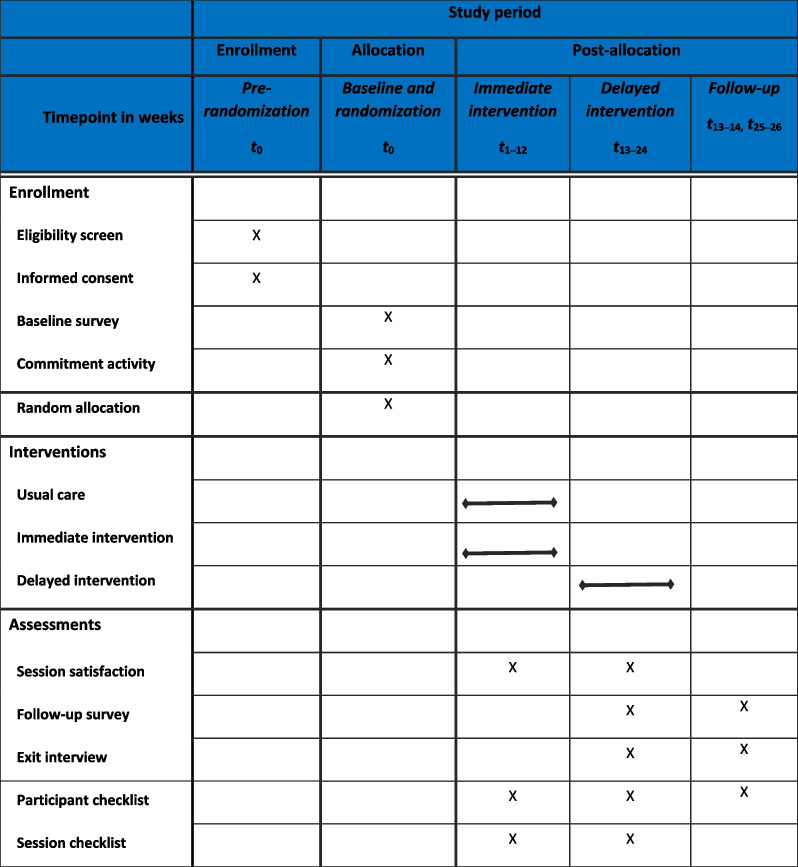
Schedule of enrollment, interventions, and assessments according to the Standard Protocol Items: Recommendations for Intervention Trials (SPIRIT) diagram

t_1__–26_ denotes months since baseline and randomization

### Allocation

The staff members enrolled participants following completion of the baseline survey and prior to the assignment of the experimental or control condition. The project director or PI who are not directly involved in recruitment, intervention implementation, or outcome assessment used computer-generated allocation sequence developed by a data manager and assigned participants to the experimental or control condition. Participants were randomized in a 1:1 ratio.

### Blinding

A fully masked design was not possible given that participants and staff members knew which intervention group they had been assigned. To mask the senior statistician who will conduct the quantitative analyses, the following strategies will be used: (1) the PHEs will deliver the intervention, and staff members will conduct all assessments, (2) participants are asked not to disclose their group assignment to the staff member conducting the assessments, and (3) the PIs will provide a masked dataset to the senior statistician so that all statistical analyses can be performed without them knowing the group assignments. Feasibility outcome data obtained for the experimental and control intervention groups using session and participant checklists cannot be masked and will be assessed and analyzed by the PIs and study team.

### Adherence to the intervention

Adherence to intervention is assessed using measures of frequency and number of intervention sessions completed, intervention completion rate, intervention dropout (with reasons), and extent to which PHEs delivered intervention as intended. This data is collected through daily record forms completed by PHEs and mentors. The PIs also have regular meetings with the PHEs and mentors to gather additional information related to intervention fidelity and acceptability, including which content/activities were covered in the session, what elements went well, what they would consider changing, and what challenges they experienced.

### Data collection

Our data collection includes surveys to assess intervention satisfaction, as well as PrEP use among participants not living with HIV and viral load among participants living with HIV. We assess HIV outcomes (i.e., engagement in HIV prevention and care), hypothesized mediators (e.g., economic improvement, gender affirmation), and background variables.

### Qualitative evaluation

We will interview all *SeCuRE* participants (*n* = 39), PHEs (*n* = 4), and mentors (*n* = 8) to identify components that were most successful and those that need refinement. To ensure intervention experiences can be recalled accurately, enrollment of participants for interviews will be on a rolling basis, with each selected participant being interviewed within 1 month of intervention completion. Implementation staff and mentors will be eligible for interviews after completing delivery of services to all assigned participants. Each interview will last between 30 and 45 min and will be held in a private room at the Ruth Ellis Center or via videoconferencing. Participants will be asked to describe the kinds of barriers they were facing as they entered the program, to discuss the interactions they had with their PHEs or mentor, and to describe how (if at all) their economic, gender affirmation, and HIV needs changed in response to the intervention components. Participants will be asked questions about the microgrant (e.g., how funds were spent, how that was helpful, areas for improvement). PHEs and mentors will be asked in their interviews to think of successful and challenging intervention participants, to reflect on why they classified them as such, and to describe the interactions they had with them and the efforts made to address their needs.

### Data analysis and presentation

CONSORT reporting guidelines will be used to report outcomes from the trial. To assess feasibility, we monitor rates of outreach, recruitment, eligibility, enrollment, session attendance, retention, and assessment completion. PHEs complete structured intervention logs after each session to assess fidelity to intervention, time needed, and feasibility of delivering the interventions as designed. Feasibility benchmarks include recruiting 5–6 participants per month, enrolling at least 80% of eligible participants into the study, participants attending at least 70% of the group and mentor sessions, and retaining at least 80% of participants in the study (i.e., completing all surveys and the exit interview). Finally, feasibility also includes PHEs completing at least 90% of structured intervention fidelity checklist logs. To assess acceptability, we modified previous intervention satisfaction evaluation surveys currently being used in our study with trans women of color and implemented at the end of each session [64]. We also assess acceptability using data on participants’ reactions to various program components gathered from the intervention logs (e.g., challenges, dislikes). Additionally, acceptability will be determined by at least 80% of participants deeming the *SeCuRE* intervention as acceptable in the immediate follow-up survey and exit interview.

We will also assess engagement in HIV prevention and care (defined as PrEP use and > 80% adherence among those not living with HIV and suppressed HIV viral load among those living with HIV collected via self-report). In line with recommendations by NIH and the research methods literature, given the sample size and intent of the pilot study, we will not conduct a formal test of outcomes or attempt to obtain an estimate of effect size [65–78]. These analyses will be conducted as a feasibility check to ensure that we assess all measures required to construct hypothesized outcomes, moderators, and mediators for a larger formal RCT.

To fulfill this primary objective, frequency tables for all variables and measures of central tendency and variability for continuous variables will characterize the sample and quantify the intervention feasibility and acceptability outcomes. The proposed analyses will be conducted using validated algorithms in the general purpose statistical programs SAS or Stata. All statistical software programming code will be fully documented to enable future code review, transparency, and results reproducibility.

We will also examine the preliminary effects of the intervention on the primary HIV status-neutral prevention and care engagement outcome, and proportions of the outcome will be plotted by group over time to describe overall patterns of change across time in the *SeCuRE* intervention group and the UC control group. Hypothesis testing will be de-emphasized in line with NIH [79] and research methods literature cautions [80, 81] regarding the instability of inferential results from small-scale pilot studies. With that caveat, we anticipate that following the *SeCuRE* intervention, intervention participants will exhibit higher odds of the HIV prevention and care outcome relative to control group participants in a time-averaged comparison of the post-baseline intervention and control groups’ outcome proportions. This exploratory comparison will be performed at alpha = 0.05 in a generalized linear mixed model (GLMM) suitable for this binary outcome. Repeated observations from each participant will be the unit of analysis.

Our team has extensive experience in retaining trans women of color in research studies. Much of our success can be attributed to our community-led approach to research efforts, specifically our collaboration with TSoCP. Using these methods, our prior studies with trans women of color have regularly yielded follow-up rates above 80–85%. We will apply our rigorous multipronged approach to ensure high rates of follow-up. Research staff are trained to emphasize several times throughout the enrollment process that it is critical that we be able to reach participants for follow-up. Participants will provide multiple forms of contact information after consenting to participate. Research staff will inquire about and update any changes to contact information at each study visit. We will also request that participants inform study staff if any of their contact information changes. Participants will be told that it is important that we reach them to see how they are doing, regardless of whether they respond to the intervention components, whether they engage in HIV prevention or care services, or whether they are doing well, that their participation in the study could increase scientific knowledge about whether this program is meaningful to the community, that their research information will be confidential, and that we are grateful for their contribution to the study. At the beginning of the study, participants are provided with an overview of all study activities, which includes their approximate follow-up dates and the incentive amount of each activity. Participants receive US $40 for the baseline survey, US $40 for the first follow-up survey, US $50 for the second follow-up survey, US $30 for the exit interview, and a US $50 bonus for completing all surveys and the exit interview. Additionally, participants are provided with transportation and a meal for each group session and receive US $20 for completing a brief satisfaction survey after each group session. Follow-up status and challenges are routinely reviewed at weekly team meetings. If follow-up rates fall, we will use a proactive management approach to identify the root source of the challenges and troubleshoot potential solutions, including holding sessions at alternate days and times and considering hybrid options for those who are not able to do the group sessions in-person. Additionally, this may include meetings with our community advisory board to brainstorm solutions or modifying procedures.

### Data management and security

The project includes both qualitative and quantitative data. We use Qualtrics-programmed surveys (e.g., electronic informed consent, baseline survey, group session feedback survey, post-program survey, and 3-month follow-up survey). Participant personal identifiable information is stored in Ripple (www.ripplescience.com), a secure HIPAA-compliant web application designed for the storing and management of personally identifying information of research participants. Ripple was initially developed at the University of Michigan to provide a user-friendly, secure, online interface where research teams can centralize the storage and management of research participants’ personal information, including name, study ID, demographics, and study workflow (e.g., group session appointments, date/amount of microgrant fund receipt, date of emergency assistance fund receipt). This information is kept in a fully encrypted format within segregated database servers compliant with HIPAA-stipulated maintenance, regulation, and security protocols. Only IRB-approved University of Michigan study staff have access to the Ripple and Qualtrics accounts where raw survey responses are stored. In-depth exit interviews will be audio-recorded using an external recording device. After the interview, the audio file will be immediately uploaded to a restricted access password-protected folder on a HIPAA-compliant University of Michigan Dropbox team folder and deleted from the physical recording device. Audio files will be transcribed and then reviewed for accuracy, with any identifying information removed from the transcript. The audio files will be retained until the interviews are thematically analyzed, within 1 year of completion of data collection, at which point they will be deleted from restricted access password-protected folder. All data will be reviewed by the PI or project director to ensure accuracy and completion.

### Data monitoring

We take the following additional steps to protect subjects from the risk of a breach in confidentiality: (1) All project staff sign a confidentiality agreement requiring them to keep private the information obtained in this study; (2) with the exception of the eligibility screener survey, the electronic informed consent, and the fund disbursement documentation, all other collected data is only identified and linked via a study ID number and remain separate — no surveys or other data that we collect contains identifying information; (3) any hard copy materials are stored in a locked file cabinet in the office of the contact PI or the project director (as appropriate); and (4) only aggregate data that cannot be used to identify individuals will be included in any reports released to other agencies or for publication. Personally identifiable information linking participants to their study ID number is stored in Ripple, separate from survey data stored in Qualtrics servers and interview data stored in restricted access password-protected folder that is only demarcated with the participants’ study ID number. Participant identifiable information will be retained for 6 months after completion of the study. After this point, all identifiable information except for zip code will be deleted from the Ripple participant software leaving only the study ID as the linking criteria for all other collected data related to an individual participant. Data coded by study ID that has been unlinked to participant identifiable information will be retained for secondary analyses, as well as recordkeeping purposes per NIH grantee requirements for 3 years. We will retain name and contact information (unlinked to any participant data) for participants who consented to being contacted for future research studies, as well as individuals who screen ineligible and who indicated they would like to be contacted about future research studies, in an Excel file stored in a university-hosted Dropbox folder, separate from study data, where access is limited to the contact PI and IRB-approved study staff.

All study staff are required to report adverse events and social harms that may be associated with receipt of the interventions and to report severe adverse events (such as death, impairment, disability, hospitalization, or any life-threatening event) to both study PIs. The PIs will report all adverse events and severe adverse events through an adverse event report to the University of Michigan Institutional Review Board (IRB) within 48 h of receiving the notification or observing the event. A summary of the adverse events and severe adverse events that occurred during the year will be included in the annual progress report to the study’s funder. Because the interventions are associated with minimal risk to participants, a data monitoring committee that is independent from the sponsor was not deemed necessary for this pilot study.

### Study governance

The project management team consists of two principal investigators (PIs), co-investigators (Co-Is), peer health educators (PHE), mentors, and research staff. The PIs meets with the PHEs and research staff weekly, and the whole team meets bimonthly to discuss the progress and management of the progress. The project has been reviewed and approved by Research Ethics Committee at the University of Michigan (HUM00239691) to ensure it meets ethical approval, and the University of North Carolina-Chapel Hill (23–3058) has entered into a reliance agreement for the University of Michigan to serve as the IRB on record. Any protocol modifications will be submitted to the University of Michigan IRB. We also have a community advisory board of trans women of color who provide their expertise in recruitment, intervention content, survey instruments, and dissemination activities.

### Dissemination policy

We are committed to disseminating the outcomes of this pilot trial in the form of publications authored by the study team in peer-reviewed journals, the ClinicalTrials.gov registry, community partner meetings, local healthcare organizations, and scientific conferences. All study findings will be shared with participants. We will compile structured de-identified datasets to be publicly available for additional/secondary data analyses that will deposited at the Inter-university Consortium for Political and Social Research (ICPSR) located at the University of Michigan.

## Discussion

This pilot study will provide important insights into the potential of microeconomic strategies to address HIV inequities among trans women of color. Beyond trans-led efforts, current microeconomic interventions often omit trans women of color. The current project builds upon existing community-led approaches to addressing economic vulnerability and has the potential to promote employment and income generation to mitigate the deleterious sequelae of racism and transphobia, improve gender affirmation, and encourage uptake and retention in HIV prevention and care, such as HIV testing and uptake of PrEP/ART.

Notably, our team has also adopted new ways of engaging with participants while minimizing physical contact during the COVID-19 pandemic using online and phone-based platforms [27]. For example, we have been successful in using videoconferencing to conduct focus groups and in-depth interviews in Detroit, MI, US. Additionally, TSoCP continued their programming at the Ruth Ellis Center delivered over videoconferencing for trans women of color during the COVID-19 pandemic. While challenging to meaningfully engage trans women of color in online interventions, our team is prepared to include relevant COVID-19 mitigation protocols (i.e., masks, social distancing) and integrate phone or online activities with the proposed in-person design as needed to mitigate COVID-19 transmission.

Results from this pilot study will provide important preliminary findings on feasibility and acceptability to inform a full-scale R01-funded trial and inform the growing literature in health promotion to enable trans women of color to realize full health and well-being. The mixed-methods approach will allow us to explore PHEs’, mentors’, and participants’ experiences and acceptability of the intervention. The findings of this pilot trial will be of relevance to policy-makers, practitioners, and researchers interested in microeconomic interventions to address economic vulnerability and HIV prevention and care outcomes among trans women of color.

### Trial status

Recruitment began in June 2024 and was completed by the end of July 2024. Immediate post-intervention assessments are expected to be completed by November 2024, the second follow-up assessments completed by February 2025, and the delayed post-intervention exit interviews completed by June 2025.

## Supplementary Information

Supplementary Material 1. SPIRIT 2013 Checklist: Recommended items to address in a clinical trial protocol and related documents.

## Data Availability

Not applicable.

## Abbreviations

AE: Adverse event
ART: Antiretroviral therapy
CAB: Community Advisory Board
CBO: Community-based organization
Co-I: Co-investigator
GLMM: Generalized linear mixed model
HIV: Human immunodeficiency virus
IRB: Institutional Review Board
MLE: Maximum likelihood model
NIH: National Institutes of Health
PHE: Peer Health Educator
PHI: Protected health information
PI: Principal investigator
PrEP: Pre-exposure prophylaxis
RCT: Randomized controlled trial
SAE: Serious adverse event
SeCuRE: Strengthening Community Responses to Economic vulnerability
TSoCP: Trans Sistas of Color Project
